# The complete mitochondrial genome and phylogenetic position of the tubeworm *Serpula uschakovi* Kupriyanova 1999 (Annelida: Serpulidae)

**DOI:** 10.1080/23802359.2026.2677264

**Published:** 2026-05-25

**Authors:** Qinghua Bao, Renyu Zheng, Elena K. Kupriyanova, Yanan Sun

**Affiliations:** a Laboratory of Marine Organism Taxonomy and Phylogeny, Shandong Province Key Laboratory of Marine Biodiversity and Bio-resource Sustainable Utilization, Institute of Oceanology, Chinese Academy of Sciences, Qingdao, China; b University of Chinese Academy of Sciences, Beijing, China; c Australian Museum Research Institute, Australian Museum, Sydney, Australia; d Department of Biological Sciences, Macquarie University, Sydney, Australia

**Keywords:** Sabellida, calcareous tubeworm, phylogeny, gene rearrangement

## Abstract

Mitochondrial genomes of the annelid family Serpulidae are characterized by a high variation in nucleotide divergence and gene arrangements. However, mitogenomic data from the genus *Serpula* remain unavailable. We report the complete mitochondrial genome of *Serpula uschakovi* from the Sea of Japan. The circular mitogenome is 20,628 bp in length, containing 13 protein-coding, two rRNA, 22 tRNA genes, and nine large non-coding regions. Its gene order exhibits extensive rearrangements when compared to the putative Pleistoannelida pattern yet retains a highly conserved 10 PCGs block shared with the genus *Hydroides*. Phylogenetic analysis placed *S. uschakovi* as the sister group to *Hydroides*.

## Introduction

Serpulidae Rafinesque, 1815, commonly known as ‘calcareous tubeworms’, is a distinctive group of the sedentary marine annelids characterized by self-secreted calcareous tubes and colorful radiolar crowns. The type species of the type genus, *Serpula vermicularis* Linnaeus, 1767, has long been considered cosmopolitan, being indiscriminately reported from tropical, subtropical, Arctic and Antarctic waters (Kupriyanova 1999). However, modern studies have demonstrated that it as a complex of morphologically similar species with restricted geographic ranges (Kupriyanova and Rzhavsky 1993; Kupriyanova 1999). Before *S. uschakovi* was described (Kupriyanova 1999), the species was reported as *S. columbiana* Johnson, 1901 and later synonymized with *S. vermicularis* by Uschakov (1955). Subsequent molecular validation confirmed its distinct status (Kupriyanova et al. 2008), illustrating the ongoing effort to resolve hidden diversity within the complex.

Mitogenomes of Serpulidae exhibit pronounced sequence divergence and frequent gene order rearrangements relative to other annelids (Seixas et al. 2017; Sun et al. 2021; Kobayashi et al. 2023). This structural plasticity represents a key family-level trait with high phylogenetic utility for assessing lineage-specific evolution (Struck et al. 2023). However, mitogenomic resources for the name-bearing type genus *Serpula* remain unavailable, despite its role as the evolutionary and nomenclatural baseline for polarizing gene order changes within the Serpulidae. Here, we report the complete mitogenome of *S. uschakovi*. The newly sequenced mitogenome adds to the growing pool of mitogenome resources for marine annelids, providing an essential genomic baseline for further species identification, phylogenetic reconstruction, and the investigation of correlations between lineage-specific rearrangement patterns and phylogenetic divergence across Serpulidae.

## Materials and methods

**Figure 1.**
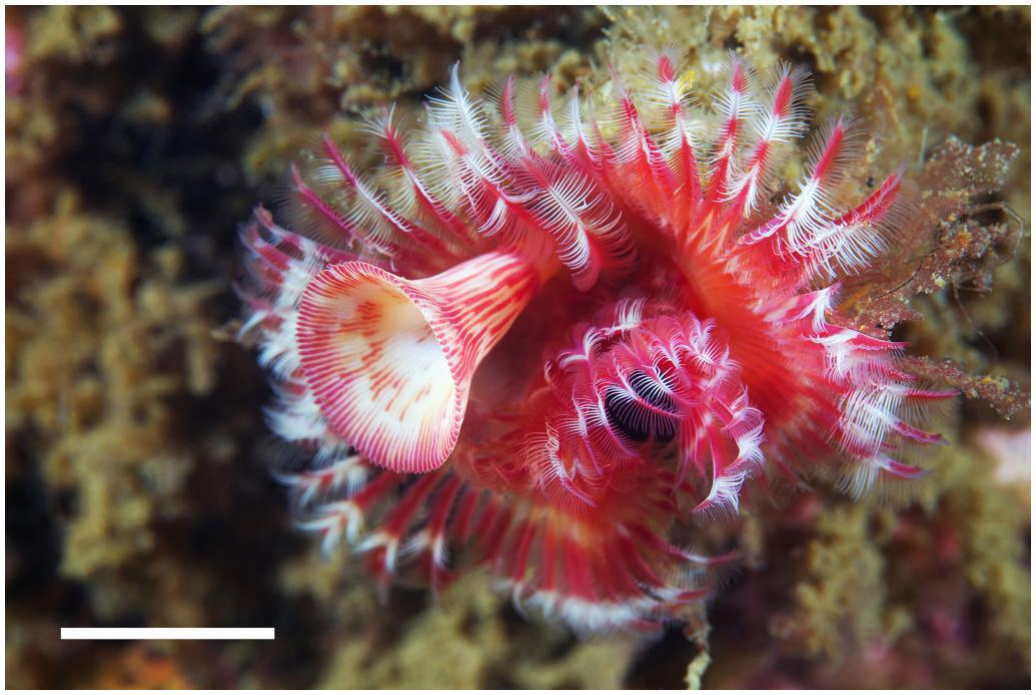
Species reference image of *Serpula uschakovi* collected from the Sea of Japan. Photograph was taken by Alexander Semenov. Scale bar: 1 cm.

A specimen of *Serpula uschakovi* was collected from its type locality, Vostok Bay, Russia (42°53′34ʺN, 132°43′48ʺE) on 14 July 2013 (Figure 1). Identification was confirmed using key morphological traits from Kupriyanova (1999) (Details in the Supplementary Methods). The specimen was fixed and preserved in 99% ethanol and deposited at the Australian Museum, Sydney, Australia (AM W.43525, contact: Claire Rowe, Claire.Rowe@Australian.Museum).

Total genomic DNA was extracted using the DNeasy^®^ Blood and Tissue Kit (Qiagen Inc., Valencia, CA). Library preparation and sequencing were performed on an MGI-T7 platform (150 bp paired-end) by Beijing Berry Genomics. Raw reads were quality filtered with fastp v0.20.0 (Chen et al. 2018) and trimmed with Trimmomatic v.0.39 (Bolger et al. 2014). The mitogenome was initially assembled using MitoFinder v.1.4.1 (Allio et al. 2020) and circularized with NOVOPlasty v.4.3.5 (Dierckxsens et al. 2017). Coverage depth was calculated using SAMtools v1.19.2 (Danecek et al. 2021) (Figure S1). Annotation was performed using MITOS2 v.2.1.8 (Donath et al. 2019) and ARWEN v1.2 (Laslett and Canbäck 2008). The *atp8* gene was identified using HMMER v.3.0 (Finn et al. 2011). Gene boundaries were manually refined with MultAlin (Corpet 1988). The circular genome map was generated using Proksee (Grant et al. 2023). AT and GC skew were calculated according to Perna and Kocher (1995). Gene order distances were calculated using CREx (Bernt et al. 2007) and the Compute distance matrix tool on the EU Galaxy server. Secondary structure of the largest non-coding region was predicted using RNAfold v2.6.3 (Lorenz et al. 2011) (Figure S2).

Phylogenetic relationships were reconstructed from amino acid sequences of the 13 PCGs from *S. uschakovi* and 16 serpulids retrieved from NCBI GenBank (full annotations in Supplementary File 1), using *Protula* sp. OX457035 and *Salmacina stellaebayensis* PX168857 (Cremer et al. 2026) as outgroups. Sequences were aligned using MAFFT v7.505 (Katoh and Standley 2013), trimmed with trimAl v.1.2 (Capella-Gutiérrez et al. 2009), and then concatenated in PhyloSuite v2 (Zhao et al. 2025). Phylogenetic reconstructions were performed using both maximum likelihood (ML) and Bayesian inference (BI) frameworks. The optimal partitioning scheme and best-fit substitution models were selected using ModelFinder v3.0.1 (Kalyaanamoorthy et al. 2017) (Table S1). ML analyses were conducted using IQ-TREE v3.0.1 (Wong et al. 2026) with 1000 ultrafast bootstrap replicates (Hoang et al. 2018). BI was conducted using MrBayes v.3.2.7a (Ronquist et al. 2012) with two parallel runs of four chains until split frequencies converged < 0.01 and the first 10% discarded as burn-in.

Detailed parameters were provided in Supplementary Methods.

## Results

**Figure 2.**
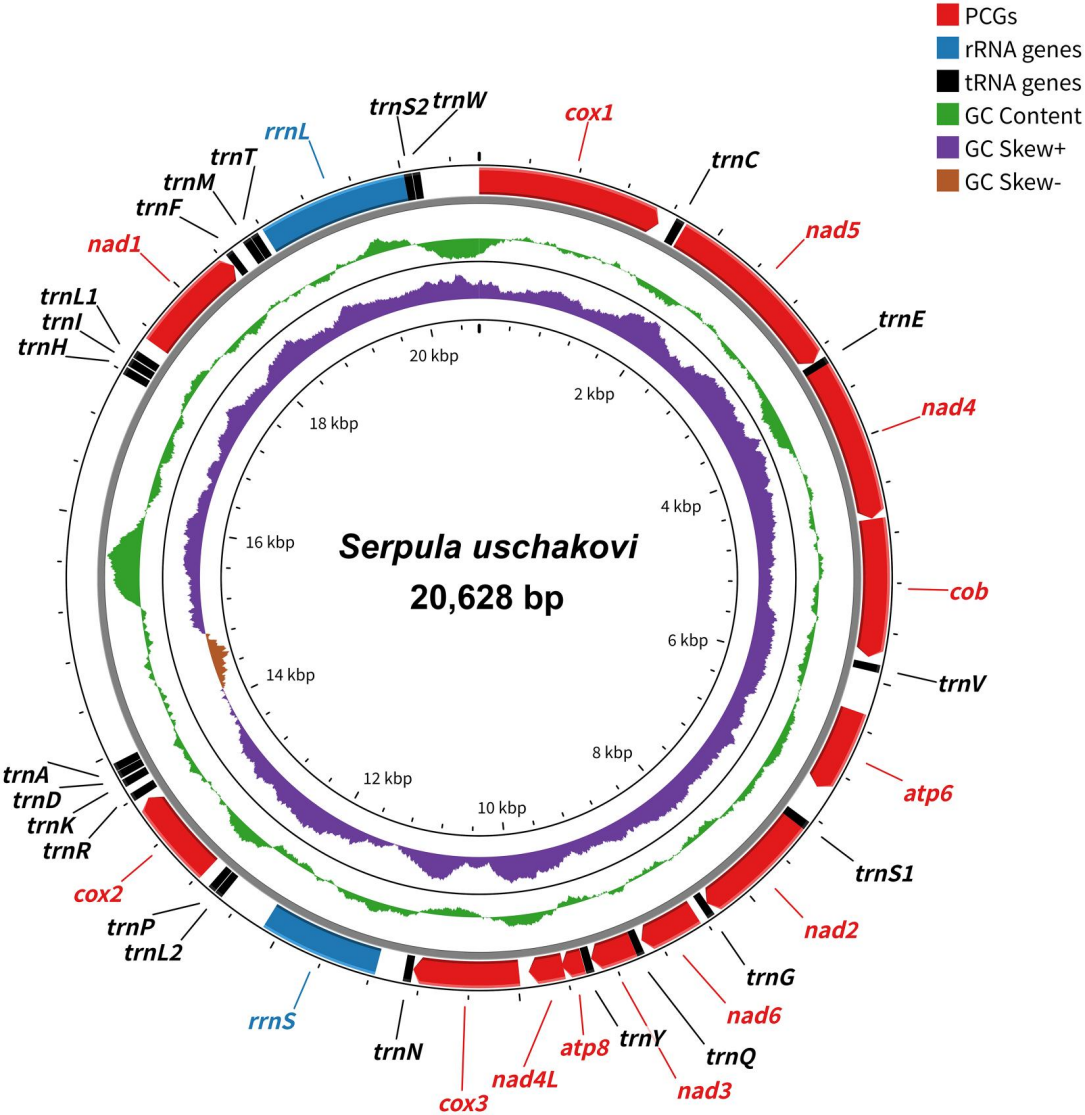
Circular map of the complete mitochondrial genome of *Serpula uschakovi*. Circles from the outside to the inside represent: (1) Gene features are shown in red (PCGs), blue (rRNA genes), and black (tRNA genes); (2) GC content (green); and (3) GC skew, with negative values shown in purple and positive values in brown. GC content and GC skew were calculated using a sliding window of 500 bp with a step size of 1 bp.

The circular mitogenome of *S. uschakovi* was 20,628 bp (GenBank accession no. PX957250). Nucleotide composition was A (24.7%), T (34.5%), G (25.0%), and C (15.8%), showing a distinct A + T bias (59.2%). AT skew was negative (−0.17), and GC skew was positive (0.23). The mitogenome comprised 37 genes (13 PCGs, 22 tRNA genes, and 2 rRNA genes) and nine large non-coding regions (>100 bp), including one 3,251 bp putative control region (Figure 2). All genes were encoded on the heavy strand. Seven PCGs initiated with start codon ATG, with ATT (*cox1*, *nad5*), ATA (*cox2*, *cox3*), and GTG (*atp6*, *nad4L*) as alternatives. Nine PCGs terminated with complete stop codons (TAA or TAG), whereas four (*atp6*, *cox1*, *cox2*, and *nad3*) featured truncated T/TA. A/T-ending codons were consistently overrepresented (RSCU > 1) across all PCGs (Table S2).

**Figure 3.**
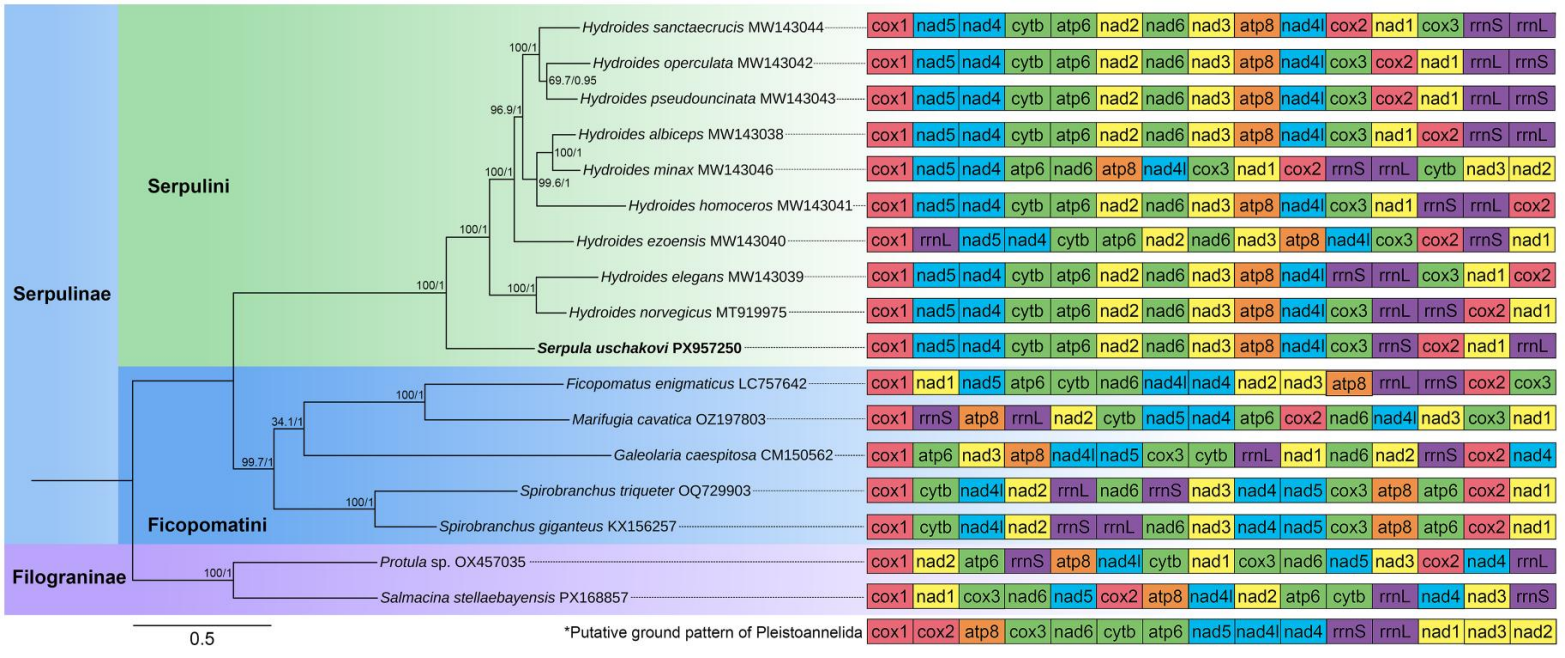
Phylogenetic relationships and mitochondrial gene arrangement of *Serpula uschakovi* and related species, inferred from maximum likelihood (ML) and Bayesian inference (BI) based on concatenated amino acid sequences of 13 mitochondrial protein-coding genes (PCGs). Tree topology shown is from the ML analyses, the BI tree was congruent. Nodal support values are UFBoot/posterior probabilities (PP). Colored gene blocks denote conserved clusters relative to the putative pleistoannelid ground pattern (PGP, asterisk). The following sequences were used: *Ficopomatus enigmaticus* LC757642 (Kobayashi et al. 2023); *Galeolaria caespitosa* CM150562 (Van Dorssen et al. 2025); *Hydroides albiceps* MW143038, *Hydroides dirampha* MW143045, *Hydroides elegans* MW143039, *Hydroides ezoensis* MW143040, *Hydroides homoceros* MW143041, *Hydroides minax* MW143046, *Hydroides norvegica* MT919975, *Hydroides operculata* MW143042, *Hydroides pseudouncinata* MW143043, *Hydroides sanctaecrucis* MW143044 (Sun et al. 2021); *Marifugia cavatica* OZ197803 (unpublished); *Protula* sp. OX457035 (unpublished); *Salmacina stellaebayensis* PX168857 (Cremer et al. 2026); *Serpula uschakovi* PX957250 (this study); *Spirobranchus giganteus* KX156257 (Seixas et al. 2017); *Spirobranchus triqueter* OQ729903 (Struck et al. 2023).

The gene order of PCGs in *S. uschakovi* was consistent with the pattern of *Hydroides* species (Figure 3). The pairwise common interval distance of mitochondrial gene arrangement between *S. uschakovi* and other serpulids ranged from 44 (vs. *Hydroides albiceps*) to 176 (vs. *Spirobranchus triqueter*) (Table S3).

The phylogenetic analyses using the ML and BI methods recovered identical well supported topologies (Figure 3). The phylogenies fully supported a close relationship between *S. uschakovi* and the monophyletic genus *Hydroides* (UFboot/PP = 100%/1).

## Discussion and conclusions

Our study presents the first mitogenome of *S. uschakovi*, filling the gap in the mitogenome data of the type genus *Serpula*. The genome size and AT bias of *S. uschakovi* mitogenome fall within the reported ranges for the family (e.g. genome size: 15,853–25,087 bp, mean 19,352 bp; AT bias: 54–66%, mean: 59.25%). The skew pattern aligns with those of other serpulids mitogenomes (Seixas et al. 2017; Sun et al. 2021; Kobayashi et al. 2023), but contrasts with that of most Sedentaria (Sun et al. 2021; Lei et al. 2022), supporting its origin in the common ancestor of Serpulidae (Kobayashi et al. 2023).

Phylogenetic reconstruction based on 13 mitochondrial PCGs supported the placement of *Serpula* and *Hydroides* within the tribe Serpulini, and the *Ficopomatus*-*Marifugia* clade and *Spirobranchus* within Ficopomatini (Kupriyanova et al. 2023), consistent with previous multi-locus and morphological studies (Kupriyanova et al. 2006, 2023; Lehrke et al. 2007), though taxon sampling remains limited.

Despite stable gene orders in Pleistoannelida (Weigert et al. 2016), only the *cytb-atp6* block is retained from the ancestral ground pattern in sampled Serpulini (Figure 3). Most sampled Serpulini share a consistent PCG block, distinct from the arrangements seen in Ficopomatini and Filograninae, underscoring the structural plasticity within Serpulidae. However, as sampling remains biased toward *Hydroides*, these identified conserve blocks are preliminary and additional structural variation will likely be revealed with broader sampling across the family.

Additionally, we identified a 131 bp intergenic spacer with high AT content (over 70%) that exhibited a significant coverage deficiency (Figure S1), likely due to stochastic sampling bias against AT-rich templates in short-read sequencing (Aird et al. 2011). The adoption of long-read sequencing in future studies should help address this issue.

In summary, the first reported mitogenome for *Serpula* serves as a critical genetic benchmark for not only addressing taxonomic challenges within the *S. vermicularis* complex but also for phylogenetic and evolutionary studies across the entire Serpulidae.

## Supplementary Material

Supplemental materials_R1.docx

Supplementary_annotation.txt

## Data Availability

The genome sequence data that support the findings of this study are openly available in GenBank of NCBI at https://www.ncbi.nlm.nih.gov under the accession no. PX957250. The associated BioProject, SRA, and Bio-Sample numbers are PRJNA1417112, SRR37060915, and SAMN54989395, respectively.
