# Supplementary material for: The complete mitochondrial genome and phylogenetic position of the tubeworm Serpula uschakovi Kupriyanova 1999 (Annelida: Serpulidae)
Source: Mitochondrial DNA B Resour. 2026 May 25;11(6):781–5. doi: 10.1080/23802359.2026.2677264 (PMC13202650; doi:10.1080/23802359.2026.2677264)
Supplement: Supplemental materials_R1.docx [file TMDN_A_2677264_SM5046.docx]

**Supplemental materials**

The complete mitochondrial genome and phylogenetic analysis of the tubeworm *Serpula uschakovi* Kupriyanova, 1999 (Annelida: Serpulidae)

Qinghua Bao, Renyu Zheng, Elena K. Kupriyanova, Yanan Sun

Supplementary Methods

**Species identification.** The identification was confirmed through a combination of the following key morphological traits: a large body size (up to 120 mm), a white calcareous tube without peristomes, a deep funnel-shaped operculum with 62–136 radii and a diameter less than or equal to its inner depth, the absence of constriction between peduncle and operculum, and the absence of a proximal rasp on bayonet chaetae (Kupriyanova, 1999).

**Genome assembly.** Total genomic DNA was extracted using the DNeasy® Blood and Tissue Kit (Qiagen Inc., Valencia, CA). A 300–400 bp insert library was prepared and sequenced on an MGI-T7 platform with 150 bp paired-end mode by Beijing Berry Genomics. Raw reads were quality filtered with fastp v0.20.0 (Chen et al., 2018) and trimmed with Trimmomatic v.0.39 (Bolger et al., 2014). The mitogenome was initially assembled using MitoFinder v.1.4.1 (Allio et al., 2020) with a set of published serpulid mitogenomes (listed in Figure 3) as references, then circularized using NOVOPlasty v.4.3.5 (Dierckxsens et al., 2017) with the MitoFinder-assembled *cox1* gene as a seed and a *k*-mer size of 33.

**Assembly validation.** Mitogenome assembly achieved 14–24,376× read depth coverage (Figure S1) using SAMtools v1.19.2 (Danecek et al., 2021). A short intergenic region between *trnW* and *cox1* (nt 20288–20418) exhibited reduced sequencing coverage (15–60×) compared with the genome-wide average of 4,163×. This 131 bp spacer has an AT content of ~70% and contains multiple homopolymer runs. The low-depth region was validated via Jellyfish (Marçais and Kingsford, 2011) *k*-mer analysis (*k* = 21), which yielded frequencies (11–20×) congruent with the observed local read depth, confirming the structural accuracy of the assembly. The NOVOPlasty assembly used the MitoFinder-assembled *cox1* gene as a seed and a *k*-mer size of 33.

**AT and GC skew calculation.** AT and GC skew were calculated using the formulas $\text{(A-T)}/\text{(A+T)}$ and $\text{(G-C)}/\text{(G+C)}$, respectively (Perna and Kocher, 1995), with a sliding window of 500 bp and a step size of 1 bp.

***atp8* identification.** The *atp8* gene was identified using HMMER v.3.0 (Finn et al., 2011) with a custom HMM profile built from amino acid sequences of published polychaete ATP8 proteins. Candidate open reading frames were translated and screened against the profile HMM, and the highest-scoring hit with a canonical start codon and flanking tRNA genes was retained.

**Phylogenetic analysis.** Amino acid sequences of the 13 protein-coding genes from *S. uschakovi* and 16 serpulid taxa were aligned using MAFFT v7.505 (Katoh and Standley, 2013) with the auto strategy, and trimmed using trimAl v1.2 (Capella-Gutiérrez et al., 2009) with the “-automated1” option. The trimmed alignments were concatenated in PhyloSuite v2 (Zhao et al., 2025). The optimal partitioning scheme and best-fit substitution models were selected under the Bayesian Information Criterion (BIC) using ModelFinder v3.0.1 (Kalyaanamoorthy et al., 2017) (Table S1). ML analyses were conducted using IQ-TREE v3.0.1 (Wong et al., 2025) with 1,000 ultrafast bootstrap replicates (Hoang et al., 2018). BI was conducted using MrBayes v.3.2.7a (Ronquist et al., 2012) with two parallel runs of four chains for 1,000,000 generations, sampling every 1,000 generations. Convergence of the MCMC chains was assessed using Tracer v1.7.2 (Rambaut et al., 2018), with effective sample size (ESS) values > 200 for all parameters. The first 10% of samples were discarded as burn-in.

**RNA secondary structure prediction.** The secondary structure of the 3,251 bp non-coding region was predicted using RNAfold v2.6.3 from the ViennaRNA Package (Lorenz et al., 2011). The sequence was folded under the Turner 2004 energy model at 37°C with 1.021 M salt concentration. The analysis was performed with parameters -p -d2 --noLP, computing the minimum free energy (MFE) structure, partition function, and ensemble diversity. The MFE structure was visualized with base-pairing probabilities ranging from purple (low probability, 0) to red (high probability, 1). The mountain plot representation includes both structural height (number of base pairs enclosing each nucleotide position) and positional entropy (a measure of local structural flexibility). All results are presented in Figure S2.

**Gene order rearrangement analysis.** Pairwise gene order rearrangement scenarios among the 17 serpulid mitogenomes were inferred using CREx (Bernt et al., 2007) on the EU Galaxy server. Only protein-coding genes and rRNA genes were included; tRNA genes were excluded due to their high variability in position. All 15 gene orders were manually aligned to start with *cox1* before analysis. The resulting pairwise rearrangement scenarios were processed using the Compute distance matrix tool on the EU Galaxy server with common intervals selected as the distance measure to generate a pairwise gene order similarity matrix, which is provided as Table S3.

Supplementary Reference

Allio, R., Schomaker-Bastos, A., Romiguier, J., Prosdocimi, F., Nabholz, B., Delsuc, F., 2020. MitoFinder: Efficient automated large-scale extraction of mitogenomic data in target enrichment phylogenomics. Molecular Ecology Resources 20, 892–905. https://doi.org/10.1111/1755-0998.13160

Bernt, M., Merkle, D., Ramsch, K., Fritzsch, G., Perseke, M., Bernhard, D., Schlegel, M., Stadler, P.F., Middendorf, M., 2007. CREx: inferring genomic rearrangements based on common intervals. Bioinformatics 23, 2957–2958. https://doi.org/10.1093/bioinformatics/btm468

Bolger, A.M., Lohse, M., Usadel, B., 2014. Trimmomatic: A flexible trimmer for Illumina sequence data. Bioinformatics 30, 2114–2120. https://doi.org/10.1093/bioinformatics/btu170

Capella-Gutiérrez, S., Silla-Martínez, J.M., Gabaldón, T., 2009. trimAl: A tool for automated alignment trimming in large-scale phylogenetic analyses. Bioinformatics 25, 1972–1973. <https://doi.org/10.1093/bioinformatics/btp348>

Chen, S., Zhou, Y., Chen, Y., Gu, J., 2018. fastp: an ultra-fast all-in-one FASTQ preprocessor. Bioinformatics 34, i884–i890. https://doi.org/10.1093/bioinformatics/bty560

Danecek, P., Bonfield, J.K., Liddle, J., Marshall, J., Ohan, V., Pollard, M.O., Whitwham, A., Keane, T., McCarthy, S.A., Davies, R.M., Li, H., 2021. Twelve years of SAMtools and BCFtools. GigaScience 10, giab008. https://doi.org/10.1093/gigascience/giab008

Dierckxsens, N., Mardulyn, P., Smits, G., 2017. NOVOPlasty: *de novo* assembly of organelle genomes from whole genome data. Nucleic Acids Research 45, e18. https://doi.org/10.1093/nar/gkw955

Finn, R.D., Clements, J., Eddy, S.R., 2011. HMMER web server: Interactive sequence similarity searching. Nucleic Acids Research 39, W29–W37. https://doi.org/10.1093/nar/gkr367

Hoang, D.T., Chernomor, O., von Haeseler, A., Minh, B.Q., Vinh, L.S., 2018. UFBoot2: Improving the ultrafast bootstrap approximation. Molecular Biology and Evolution 35, 518–522. https://doi.org/10.1093/molbev/msx281

Kalyaanamoorthy, S., Minh, B.Q., Wong, T.K.F., von Haeseler, A., Jermiin, L.S., 2017. ModelFinder: Fast model selection for accurate phylogenetic estimates. Nature Methods 14, 587–589. https://doi.org/10.1038/nmeth.4285

Katoh, K., Standley, D.M., 2013. MAFFT multiple sequence alignment software version 7: Improvements in performance and usability. Molecular Biology and Evolution 30, 772–780. https://doi.org/10.1093/molbev/mst010

Lorenz, R., Bernhart, S.H., Höner Zu Siederdissen, C., Tafer, H., Flamm, C., Stadler, P.F., Hofacker, I.L., 2011. ViennaRNA package 2.0. Algorithms Mol Biol 6, 26. https://doi.org/10.1186/1748-7188-6-26

Marçais, G., Kingsford, C., 2011. A fast, lock-free approach for efficient parallel counting of occurrences of *k* -mers. Bioinformatics 27, 764–770. https://doi.org/10.1093/bioinformatics/btr011

Perna, N.T., Kocher, T.D., 1995. Patterns of nucleotide composition at fourfold degenerate sites of animal mitochondrial genomes. Journal of Molecular Evolution 41, 353–358.https://doi.org/10.1007/BF00186547

Rambaut, A., Drummond, A.J., Xie, D., Baele, G., Suchard, M.A., 2018. Posterior summarization in Bayesian phylogenetics using Tracer 1.7. Systematic Biology 67, 901–904. <https://doi.org/10.1093/sysbio/syy032>

Ronquist, F., Teslenko, M., van der Mark, P., Ayres, D.L., Darling, A., Höhna, S., Larget, B., Liu, L., Suchard, M.A., Huelsenbeck, J.P., 2012. MrBayes 3.2: Efficient Bayesian phylogenetic inference and model choice across a large model space. Systematic Biology 61, 539–542. https://doi.org/10.1093/sysbio/sys029

Wong, T., Ly-Trong, N., Ren, H., Baños, H., Roger, A., Susko, E., Bielow, C., De_Maio, N., Goldman, N., Hahn, M., Huttley, G., Lanfear, R., Minh, B.Q., 2025. IQ-TREE 3: Phylogenomic inference software using complex evolutionary models. https://doi.org/10.32942/X2P62N

Zhao, D., Ye, T., Gao, F., Jakovlić, I., La, Q., Tong, Y., Liu, X., Song, R., Liu, F., Lian, Z., Zou, H., Li, W.-X., Wang, G.-T., Zeng, B., Zhang, D., 2025. PhyloSuite v2: The development of an all-in-one, efficient and visualization-oriented suite for molecular dating analysis and other advanced features. iMeta 4, e70095. <https://doi.org/10.1002/imt2.70095>


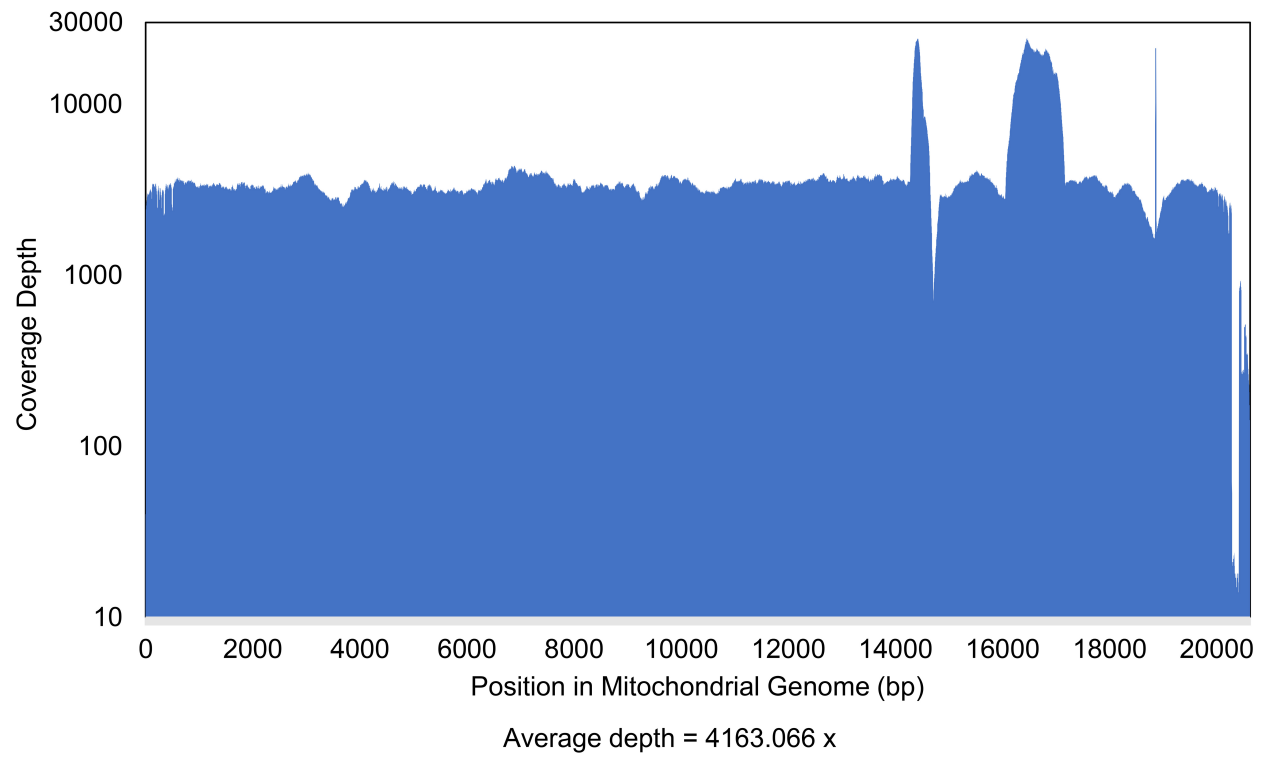


Figure S1. Sequencing depth and coverage analysis of the complete mitochondrial genome of *Serpula uschakovi*. The plot illustrates the distribution of mapped reads across the entire mitochondrial genome. The X-axis represents the nucleotide position (bp), and the Y-axis represents the depth of coverage on a logarithmic scale. The blue area indicates the sequencing depth at each position, ranging from a minimum of 14× to a peak of ~24,376×, with a mean coverage depth of 4163.066×. The continuous coverage >10× across the full length confirms the assembly integrity without gaps.


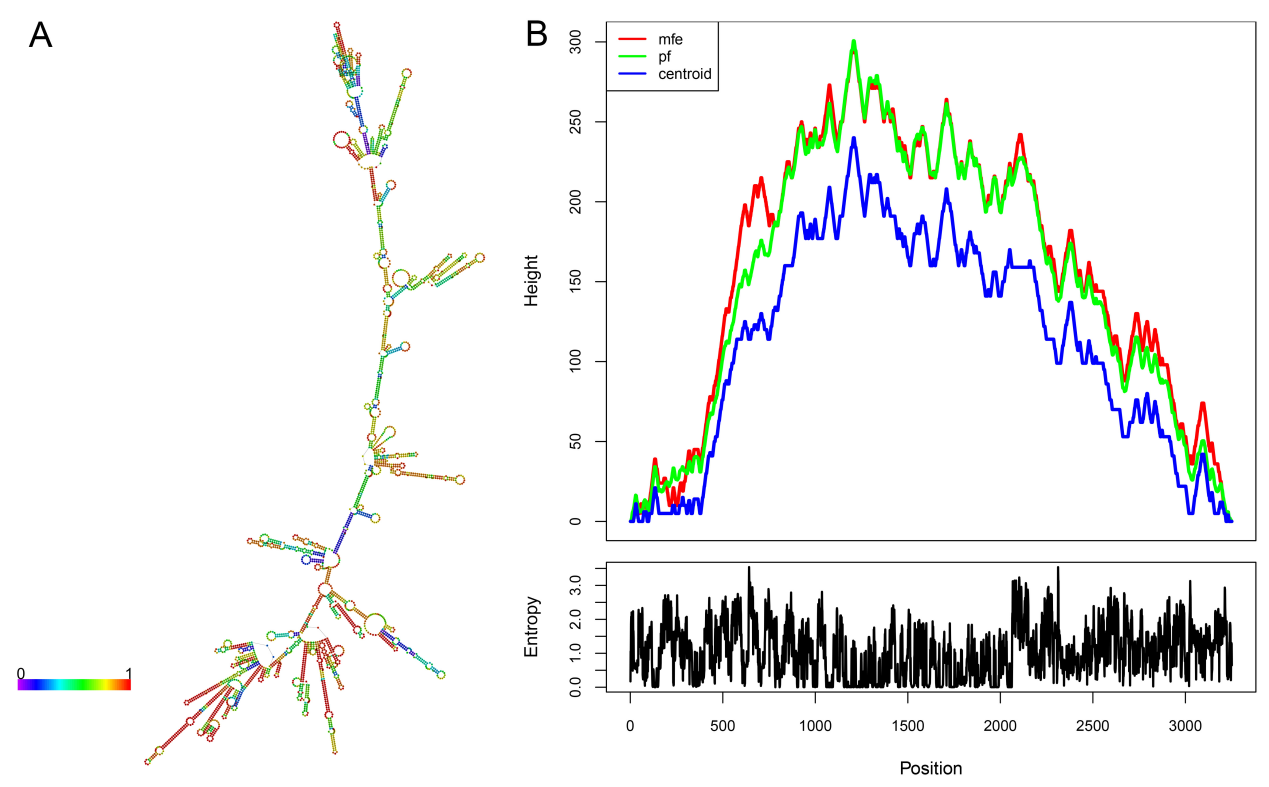


Figure S2. RNA secondary structure prediction of the 3,251 bp non-coding region in *Serpula uschakovi*. (A) Minimum free energy (MFE) secondary structure colored by base-pairing probabilities, ranging from purple (low probability) to red (high probability). (B) Mountain plot representation of the structural ensemble, showing (upper panel) the height along the sequence (number of base pairs enclosing each nucleotide position) and (lower panel) the positional entropy (a measure of structural flexibility at each position). The prominent central peak in the height panel indicates a large core structural domain, while peripheral peaks correspond to local stem-loop structures.

Table S1. Information on data partitions, amino acid lengths, and best-fit evolutionary models under the Bayesian Information Criterion (BIC) for Maximum Likelihood (ML) and Bayesian Inference (BI) analyses.

| Partition | Protein | Length (aa) | Model for ML  (IQ-TREE) | Model for BI (MrBayes) |
| --- | --- | --- | --- | --- |
| Subset1 | ATP6 | 141 | MTINV+F+I+G4 | JTT+F+I+G4 |
|  | ATP8 | 30 |  |  |
|  | COX2 | 200 |  |  |
|  | NAD2 | 273 |  |  |
|  | NAD4 | 435 |  |  |
|  | NAD4L | 75 |  |  |
|  | NAD5 | 511 |  |  |
|  | NAD6 | 122 |  |  |
| Subset3 | COX1 | 476 | MTINV+F+I+G4 | WAG+F+I+G4 |
|  | COX3 | 257 |  |  |
|  | CYTB | 359 |  |  |
|  | NAD1 | 302 |  |  |
|  | NAD3 | 109 |  |  |

Table S2. Relative synonymous codon usage (RSCU) of the 13 protein-coding genes in the *Serpula uschakovi* mitogenome. Values > 1 indicate overrepresented codons. Codon table: Invertebrate Mitochondrial (transl_table = 5).

| Codon | Count | RSCU | Codon | Count | RSCU | Codon | Count | RSCU | Codon | Count | RSCU |
| --- | --- | --- | --- | --- | --- | --- | --- | --- | --- | --- | --- |
| UUU(F) | 252 | 1.76 | UCU(S) | 83 | 2.00 | UAU(Y) | 103 | 1.45 | UGU(C) | 70 | 1.56 |
| UUC(F) | 34 | 0.24 | UCC(S) | 14 | 0.34 | UAC(Y) | 39 | 0.55 | UGC(C) | 20 | 0.44 |
| UUA(L) | 238 | 2.57 | UCA(S) | 25 | 0.60 | UAA(*) | 5 | 1.11 | UGA(W) | 72 | 0.97 |
| UUG(L) | 104 | 1.12 | UCG(S) | 11 | 0.27 | UAG(*) | 4 | 0.89 | UGG(W) | 77 | 1.03 |
| CUU(L) | 98 | 1.06 | CCU(P) | 76 | 1.99 | CAU(H) | 53 | 1.23 | CGU(R) | 37 | 1.63 |
| CUC(L) | 18 | 0.19 | CCC(P) | 28 | 0.73 | CAC(H) | 33 | 0.77 | CGC(R) | 9 | 0.40 |
| CUA(L) | 73 | 0.79 | CCA(P) | 37 | 0.97 | CAA(Q) | 29 | 1.05 | CGA(R) | 24 | 1.05 |
| CUG(L) | 24 | 0.26 | CCG(P) | 12 | 0.31 | CAG(Q) | 26 | 0.95 | CGG(R) | 21 | 0.92 |
| AUU(I) | 138 | 1.73 | ACU(T) | 58 | 1.81 | AAU(N) | 57 | 1.36 | AGU(S) | 52 | 1.25 |
| AUC(I) | 22 | 0.28 | ACC(T) | 21 | 0.66 | AAC(N) | 27 | 0.64 | AGC(S) | 15 | 0.36 |
| AUA(M) | 125 | 1.38 | ACA(T) | 35 | 1.09 | AAA(K) | 42 | 0.92 | AGA(S) | 46 | 1.11 |
| AUG(M) | 56 | 0.62 | ACG(T) | 14 | 0.44 | AAG(K) | 49 | 1.08 | AGG(S) | 86 | 2.07 |
| GUU(V) | 142 | 1.56 | GCU(A) | 110 | 2.04 | GAU(D) | 48 | 1.52 | GGU(G) | 96 | 1.09 |
| GUC(V) | 28 | 0.31 | GCC(A) | 41 | 0.76 | GAC(D) | 15 | 0.48 | GGC(G) | 37 | 0.42 |
| GUA(V) | 99 | 1.08 | GCA(A) | 33 | 0.61 | GAA(E) | 52 | 0.87 | GGA(G) | 71 | 0.81 |
| GUG(V) | 96 | 1.05 | GCG(A) | 32 | 0.59 | GAG(E) | 68 | 1.13 | GGG(G) | 147 | 1.68 |

Table S3. Matrix of pairwise gene order rearrangement distances among 17 serpulid mitogenomes. Values represent common interval distances between each species pair. Only protein-coding genes and rRNA genes were included; all 15 gene orders were manually aligned to start with cox1 before analysis. Lower values indicate higher gene order similarity. Diagonal cells (0) represent self-comparisons. Abbreviations: Fen, Ficopomatus enigmaticus; Gca, Galeolaria caespitosa; Hal, Hydroides albiceps; Hel, Hydroides elegans; Hez, Hydroides ezoensis; Hho, Hydroides homoceros; Hmi, Hydroides minax; Hno, Hydroides norvegica; Hop, Hydroides operculata; Hps, Hydroides pseudouncinata; Hsa, Hydroides sanctaecrucis; Mca, Marifugia cavatica; Psp, Protula sp.; Sst, Salmacina stellaebayensis; Sus, Serpula uschakovi; Sgi, Spirobranchus giganteus; Str, Spirobranchus triqueter.

|  | Fen | Gca | Hal | Hel | Hez | Hho | Hmi | Hno | Hop | Hps | Hsa | Mca | Psp | Sst | Sus | Sgi | Str |
| --- | --- | --- | --- | --- | --- | --- | --- | --- | --- | --- | --- | --- | --- | --- | --- | --- | --- |
| Fen | 0 |  |  |  |  |  |  |  |  |  |  |  |  |  |  |  |  |
| Gca | 176 | 0 |  |  |  |  |  |  |  |  |  |  |  |  |  |  |  |
| Hal | 166 | 170 | 0 |  |  |  |  |  |  |  |  |  |  |  |  |  |  |
| Hel | 166 | 172 | 82 | 0 |  |  |  |  |  |  |  |  |  |  |  |  |  |
| Hez | 162 | 170 | 66 | 108 | 0 |  |  |  |  |  |  |  |  |  |  |  |  |
| Hho | 168 | 172 | 46 | 66 | 88 | 0 |  |  |  |  |  |  |  |  |  |  |  |
| Hmi | 172 | 174 | 132 | 158 | 162 | 150 | 0 |  |  |  |  |  |  |  |  |  |  |
| Hno | 160 | 170 | 60 | 64 | 82 | 66 | 150 | 0 |  |  |  |  |  |  |  |  |  |
| Hop | 168 | 170 | 46 | 82 | 70 | 66 | 150 | 66 | 0 |  |  |  |  |  |  |  |  |
| Hps | 168 | 170 | 46 | 82 | 70 | 66 | 150 | 66 | 0 | 0 |  |  |  |  |  |  |  |
| Hsa | 170 | 172 | 44 | 78 | 88 | 82 | 148 | 84 | 66 | 66 | 0 |  |  |  |  |  |  |
| Mca | 170 | 180 | 170 | 170 | 166 | 170 | 172 | 170 | 170 | 170 | 170 | 0 |  |  |  |  |  |
| Psp | 180 | 176 | 172 | 172 | 174 | 174 | 174 | 176 | 176 | 176 | 172 | 176 | 0 |  |  |  |  |
| Sst | 174 | 176 | 170 | 170 | 168 | 170 | 174 | 170 | 170 | 170 | 168 | 166 | 162 | 0 |  |  |  |
| Sus | 168 | 168 | 44 | 88 | 46 | 70 | 148 | 64 | 64 | 64 | 66 | 172 | 174 | 172 | 0 |  |  |
| Sgi | 176 | 176 | 172 | 170 | 174 | 174 | 174 | 168 | 172 | 172 | 172 | 174 | 178 | 174 | 174 | 0 |  |
| Str | 178 | 178 | 176 | 174 | 176 | 178 | 176 | 172 | 176 | 176 | 176 | 172 | 178 | 170 | 176 | 46 | 0 |
